# Heterogeneity of psychological resilience among individuals with recurrent implantation failure: a latent profile analysis

**DOI:** 10.3389/fpsyt.2026.1798373

**Published:** 2026-03-20

**Authors:** Yue Peng, Yan Pu, Yuyang Wang, Shiya Li, Yan Wang, Jinbo Fang

**Affiliations:** 1 Department of Reproductive Medicine Nursing, West China Second University Hospital, Sichuan University/West China School of Nursing, Sichuan University, Chengdu, China; 2 Key Laboratory of Birth Defects and Related Diseases of Women and Children (Sichuan University), Ministry of Education, Chengdu, China; 3 West China School of Nursing, Sichuan University, Chengdu, China

**Keywords:** assisted reproductive technology, clinical decision, latent profile analysis, mental health, psychological resilience, recurrent implantation failure

## Abstract

**Objective:**

To ascertain the level of psychological resilience, examine the latent profiles of individuals within infertile couples who experience recurrent implantation failure (RIF), identify the relevant influencing factors, and lay a foundation for developing customized intervention strategies.

**Methods:**

Convenience sampling was adopted in this study. Participants were selected from individuals in infertile couples with RIF who attended the Second West China Hospital of Sichuan University between November 2024 and July 2025. Data were collected via a general information questionnaire and validated scales assessing psychological resilience, social support, sleep quality, family adaptability and cohesion, anxiety, and depression. Latent profile analysis (LPA) was performed to explore the psychological resilience profiles of individuals with RIF, while univariate analysis and multivariate Logistic regression analyses were employed to identify the influencing factors associated with different profile categories.

**Results:**

A total of 303 valid questionnaires were collected, including 194 from females and 109 from males. The overall psychological resilience score was (26.66 ± 6.319). Latent profile analysis categorized psychological resilience into three subgroups: the low tenacity-low strength subgroup (31.4%), the moderate tenacity-moderate strength subgroup (53.1%), and the high tenacity-high strength subgroup (15.5%); Multivariate Logistic regression analysis indicated that gender, family adaptability and depression severity (all *P* < 0.05) were independent predictors of psychological resilience profile membership.

**Conclusion:**

Marked interindividual heterogeneity exists in the psychological resilience of individuals with RIF. Gender, family adaptability and depression severity serve as the core influencing factors. In clinical practice, stratified and targeted interventions should be delivered according to distinct psychological resilience subgroups. It yields clinical implications for an association between improved psychological resilience among individuals from couples with RIF and enhanced treatment adherence.

## 1 Introduction

Infertility is a reproductive disorder characterized by the inability to achieve a clinical pregnancy following at least one year of regular unprotected sexual intercourse. According to the 2025 WHO Global Guideline for the Prevention, Diagnosis and Treatment of Infertility (1), approximately 1 in 6 people of reproductive age worldwide experience infertility at some point in their lifetime. Research findings demonstrate that the prevalence of infertility in China surged notably from 12% to 18% between 2007 and 2020 (2), reflecting a marked deterioration of reproductive health concerns. The diagnostic and therapeutic procedures for infertility, coupled with substantial financial burdens and social prejudice, can induce psychological distress of varying severity in affected individuals, trigger conflicts within family dynamics, exacerbate household economic strain, and significantly impair the quality of life of infertility individuals. Owing to its high prevalence, profound implications for families and society, and substantial economic burden, infertility has emerged as a pressing global public health challenge demanding immediate attention. In recent decades, assisted reproductive technology (ART) has addressed fertility challenges for an increasing number of infertile households worldwide. Assisted reproductive technology (ART) has a 30-year development history in China. As controlled ovarian stimulation protocols are continuously refined and embryo laboratory techniques and conditions are progressively optimized, the success rate of *in vitro* fertilization-embryo transfer (IVF-ET) has witnessed a steady rise. Nevertheless, a notable proportion of individuals still encounter recurrent implantation failure (RIF), which is defined as the absence of clinical pregnancy following the transfer of at least 4 high-quality cleavage-stage embryos or 2 blastocysts over a minimum of 2 fresh or frozen-thawed cycles (3), with its prevalence estimated at around 10% (4). Individuals with RIF are likely to experience heightened psychological distress during the course of treatment (5) and exhibit an increased susceptibility to negative emotional states including anxiety and depression (6). Thus, greater attention should be directed toward the mental health of the RIF population.

The present study concentrates on the psychological resilience traits of individuals within the RIF population, providing an individual-level basis for future couple-based dyadic resilience investigations.As a positive trait and source of strength that embodies patient growth in the field of positive psychology, psychological resilience exerts a vital role in fostering mental health among populations affected by recurrent implantation failure. Research evidence indicates that robust psychological resilience serves as a pivotal factor enabling individuals to effectively navigate the adversities associated with infertility; it facilitates the tolerance of infertility-related stress, promotes recovery from negative emotional states, acts as a critical protective factor against detrimental psychological outcomes including anxiety and depression, and functions as a positive predictor of subjective well-being (7, 8). Dyadic resilience theory emphasizes that interaction and collaborative coping between couples are important sources of resilience (9), while family system theory points out that family adaptability and cohesion are core dimensions of family-level protective factors (10). Previous studies have suggested that family system functioning exerts a crucial regulatory association with psychological resilience among individuals with infertility (11). Latent Profile Analysis (LPA) is an individual-oriented research approach that uncovers latent trait classifications and heterogeneity among study participants by identifying their response patterns across observable manifest variables (12). Thus, the present study employed the LPA approach to investigate the latent subgroups and factors associated with psychological resilience among individuals from couples with recurrent implantation failure (RIF). This research is anticipated to furnish critical evidence for the development of targeted intervention strategies, and holds substantial clinical practical significance for mitigating negative emotions, enhancing treatment adherence, and improving reproductive quality of life in this population.

## 2 Methods

### 2.1 Study design and participants

Individuals from infertile couples with RIF who attended the Second West China Hospital of Sichuan University between November 2024 and July 2025 were consecutively recruited as study participants using a convenience sampling method. Inclusion criteria (1): Meeting the diagnostic criteria for RIF: individuals have experienced at least 2 fresh or frozen-thawed cycles, with a total transfer of no less than 4 high-quality cleavage-stage embryos or 2 blastocysts, without attaining clinical pregnancy; (2) Being mentally clear, having a certain level of comprehension and reading ability, being able to independently complete the questionnaire content, and clearly expressing inner feelings; (3) Voluntarily agreeing to participate in the study and signing the informed consent form. (4) Mentally alert, with normal vision, sufficient comprehension and literacy, and no physical or psychiatric conditions affecting questionnaire fulfillment; (5) No history of major psychological trauma, psychiatric disorders, or intellectual impairment. Exclusion criteria: (1) Having psychiatric disorders, intellectual impairment, visual deficits, or other conditions that hinder independent questionnaire completion; (2) Coexistence of serious physical illnesses including malignant neoplasms and severe organ complications; (3) Currently enrolled in other clinical trials that could potentially influence the outcomes of this study.

### 2.2 Sample size

For statistical variable analysis, the required sample size is generally 10 times the number of influencing factors. Based on an extensive review of domestic and international literature, the suspected influencing factors of psychological resilience were initially identified. After literature review, 19 influencing factors were predicted, including: social support, family support, general self-efficacy, coping styles, anxiety and depression status, fertility-related stress, age, pregnancy history, ethnicity, religious belief, duration of infertility diagnosis, type of infertility, educational level, occupation, monthly family income, long-term place of residence, total treatment cost incurred, duration of treatment, and marital quality. Therefore, the sample size of this study is set at 95-190. Considering a 20% loss to follow-up rate, the actual required sample size should be 114-228 cases. Latent profile analysis requires a valid sample size of at least 200 cases and no fewer than 10 times the number of latent profiles. Nylund−Gibson et al. (13) recommended in their practical guidelines that an ideal sample size should be at least 300. This study finally included 303 valid cases, far exceeding the 30 cases required for 3 latent profiles, and also met the requirement of more than 100 times the number of observed variables (3), which can ensure the stability of LPA model fitting and the reliability of classification results.

### 2.3 Data collection and management

The present study regarded each partner in RIF couples as independent subjects; dyadic data were not analyzed in pairs, and all data were incorporated into the overall analysis at the individual level. Independent identification numbers were assigned to both spouses during data collection to prevent data mixing and guarantee the independence of analyses at the individual level.Data collection was performed by researchers who received standardized training, following this specific procedure: Initially, potential participants were screened via medical record review and on-site assessments based on the study’s inclusion and exclusion criteria. Following a detailed explanation of the study’s purpose, content, procedures, and instructions for completing the questionnaires, potential participants were provided with a written informed consent form. They were explicitly notified of their right to voluntary participation and unrestricted withdrawal, alongside assurance that all data would be anonymized to safeguard their privacy. Upon receiving the signed informed consent forms, anonymous questionnaires were issued to the participants. The time required to complete the questionnaire was around 10 to 15 minutes. Following questionnaire completion, researchers collected and inspected the completeness and quality of the forms immediately. For any missing items, participants were prompted to fill them in.

### 2.4 Measurement tools

This study employed a self-developed general information questionnaire to collect the sociodemographic characteristics of participants, such as age, per capita monthly household income, and presence of biological children. The following standardized assessment tools were used to measure indicators including anxiety, depression, social support, family cohesion and adaptability, sleep quality, and psychological resilience.

1. Anxiety and depression: The 7-item Generalized Anxiety Disorder Scale (GAD-7) was used to assess the anxiety symptoms of the research subjects. Developed by Spitzer (14)in 2006, this scale consists of 7 items, each scored on a 4-point Likert scale ranging from 0 to 3, with a total score ranging from 0 to 21. A higher score indicates a more severe level of anxiety. He Xiaoyan et al. (15) reported that the Cronbach’s α coefficient of the GAD-7 was 0.898 and the test-retest reliability coefficient was 0.856, confirming that the Chinese version has good reliability and validity. Depressive symptoms were evaluated with the 9-item Patient Health Questionnaire (PHQ-9). The scale includes 10 items: 9 symptom-specific items and 1 global functional assessment item. Each item is rated on a 4-point scale (0-3), with a total score ranging from 0 to 27, for assessing the severity of depressive symptoms. Chen Manman et al. (16) verified that the 9-item PHQ has good validity and reliability in general outpatient populations. In this study, PHQ-9 and GAD-7 scores were dichotomized according to established clinical cut-off values (≤9 = low severity, >9 = moderate to high severity). These cut-off values have been previously validated for use among individuals with infertility (14–16).
2. Social support: The Social Support Rating Scale (SSRS) (17) was employed to measure the social support level of participants. The scale comprises 3 dimensions and 10 items, with total scores categorized into three levels: low (≤22 points), medium (23-44 points), and high (45-66 points). Higher total scores indicate higher social support levels. The Cronbach’s α coefficients of the total scale and its three subscales are 0.896, 0.849, 0.825, and 0.833, respectively. In this study, SSRS scores were dichotomized according to established clinical cut-off values (≤ 45 = low to moderate level, > 45 = high level).
3. Family cohesion and adaptability: The Family Adaptability and Cohesion Evaluation Scales II (FACES II) was utilized for assessment. Originally developed by Olson et al. in 1982, the scale was translated into Chinese by L. Fei et al. (18) Family cohesion refers to the emotional connection among family members, while adaptability denotes the ability of the family system to adjust in response to challenges from the family environment and different developmental stages. The two subscales include 30 items: 16 on family cohesion and 14 on adaptability. Responses are rated on a 5-point Likert scale (1 = Never, 2 = Seldom, 3 = Sometimes, 4 = Often, 5 = Always), with higher scores indicating greater family cohesion and adaptability. In this study, FACES II scores were dichotomized according to established clinical cut-off values (Family cohesion: ≤ 63 = low to moderate level, > 63 = high level; Family adaptability: ≤ 50 = low to moderate level, > 50 = high level).
4. Sleep quality: The Pittsburgh Sleep Quality Index (PSQI) was employed to evaluate sleep quality among participants. The scale was originally developed by Buysse et al. (19) in 1989.This tool comprises 7 dimensions (sleep quality, sleep latency, sleep duration, sleep efficiency, sleep disturbances, hypnotic use, and daytime dysfunction) and 23 items, for assessing sleep status over the past month. Each dimension is scored 0-3, with a total score ranging from 0 to 21; higher scores indicate poorer sleep quality. In this study, PSQI scores were dichotomized according to established clinical cut-off values (≤ 10 = good sleep quality, > 10 = poor to moderate sleep quality).
5. Psychological resilience: The 10-item Connor-Davidson Resilience Scale (CD-RISC-10) was employed for assessment. This scale is a shortened version of the original Connor-Davidson Resilience Scale (CD-RISC) (20).Wang et al. (21) released the Chinese version of the CD-RISC-10 in 2010. The scale includes 10 items, each rated on a 5-point Likert scale (0 = Never, 1 = Seldom, 2 = Sometimes, 3 = Often, 4 = Always). The total score is the sum of all items, ranging from 0 to 40, with higher scores indicating higher levels of psychological resilience. The scale has a Cronbach’s α coefficient of 0.91 and a test-retest reliability of 0.90, making it suitable for evaluating psychological resilience in Chinese infertile individuals.

### 2.5 Data analysis

With the scores of the three dimensions of the Psychological Resilience Scale serving as manifest indicators, latent profile models were constructed using Mplus 8.0 software, and models encompassing 1 to 5 profiles were fitted successively for model selection. Latent profile model fit was evaluated using the following indices: ① Information criteria: Akaike information criterion (AIC), Bayesian information criterion (BIC), and adjusted Bayesian information criterion (aBIC). Lower values of these three indices denote superior model fit. ② Classification index: Entropy, which ranges from 0 to 1. A larger entropy value reflects more accurate classification and thus better model fit. ③ Likelihood ratio test indices: Lo-Mendell-Rubin likelihood ratio test (LMRT) and Bootstrapped likelihood ratio test (BLRT). A P value < 0.05 signifies that the k-profile model outperforms the (k-1)-profile model. Statistical analyses were conducted with SPSS 24.0 software. Normally distributed measurement data were presented as mean ± standard deviation, and categorical data were summarized as frequencies and percentages. Differences between groups were assessed using the chi-square test or rank-sum test, as appropriate. With the latent profile classification results as the dependent variable, and variables that were statistically significant in univariate analyses as independent variables, multivariate Logistic regression analysis was conducted to identify the influencing factors. A *P* value < 0.05 was considered to indicate a statistically significant difference.

### 2.6 Ethical statement

This study was approved by Ethics Committee of West China Second University Hospital, Sichuan University (No.2024035). The purpose, content and significance of the study were explained to the participants during the investigation. We abided research process by the ethical principle of informed consent, voluntary, harmless. All study procedures were strictly followed the Declaration of Helsinki.

## 3 Results

### 3.1 Demographic characteristics and univariate analysis of latent profiles in individuals from couples with recurrent implantation failure

A total of 303 valid questionnaires were recovered in this study, including 194 females (64.0%) and 109 males(36.0%); 42 cases (13.9%) were aged ≤30 years and 261 cases (86.1%) were aged >30 years; 59 cases (19.5%) had a college diploma or below and 244 cases (80.5%) had an education level above college; 48 cases (15.8%) had a total monthly family income of ≤5000 RMB and 255 cases (84.2%) had an income of >5000 RMB; 270 cases (89.1%) had no biological children and 33 cases (10.9%) had biological children. The overall psychological resilience score of individuals with recurrent implantation failure (RIF) was (26.66 ± 6.319), among which the score of the tenacity dimension was (13.34 ± 0.199), the strength dimension was (7.80 ± 0.117), and the optimism dimension was (5.52 ± 0.081). The results showed that the distribution differences of the three latent profiles of psychological resilience in RIF individuals were statistically significant in the dimensions of gender, social support, intimacy, adaptability, anxiety and depression (all *P* < 0.05), while there were no statistically significant differences in the dimensions of age, education level, income, having biological children or not, and sleep (all *P*>0.05). Given the non-significant result of PSQI in the univariate analysis, it was not included in the subsequent multivariate Logistic regression analysis. Although SSRS showed a statistically significant difference in the univariate analysis, it did not serve as an independent influencing factor for the latent profiles of psychological resilience in the multivariate analysis. The demographic characteristics of the participants and the univariate analysis results of latent profiles are presented in **Table 1**.

**Table 1:** Demographic characteristics and univariate analysis outcomes of the three psychological resilience latent profiles among individuals from couples with recurrent implantation failure.

Characteristics		Double-low subgroup (n=95)	Double-moderate subgroup (n=161)	Double-high subgroup (n=47)	Total	*X^2^*	*P*
Gender	Male	26 (27.40%)	57 (35.40%)	26 (55.30%)	109 (36.00%)	10.714	0.005
	Female	69 (72.60%)	104 (64.60%)	21 (44.70%)	194 (64.00%)		
Age	Under 30 years	13 (13.70%)	21 (13.00%)	8 (17.00%)	42 (13.90%)	0.486	0.784
	Over 30 years	82 (86.30%)	140 (87.00%)	39 (83.00%)	261 (86.10%)		
Education	Below college education	22 (23.20%)	28 (17.40%)	9 (19.10%)	59 (19.50%)	1.271	0.53
	Above college education	73 (76.80%)	133 (82.60%)	38 (80.90%)	244 (80.50%)		
Monthly household total income	Below 5000 yuan	19 (20.00%)	21 (13.00%)	8 (17.00%)	48 (15.80%)	2.227	0.328
	Over 5000 yuan	76 (80.00%)	140 (87.00%)	39 (83.00%)	255 (84.20%)		
Biological children	No	82 (86.30%)	144 (89.40%)	44 (93.60%)	270 (89.10%)	1.766	0.413
	Yes	13 (13.70%)	17 (10.60%)	3 (6.40%)	33 (10.90%)		
SSRS	0-45 scores	91 (95.80%)	136 (84.50%)	40 (85.10%)	267 (88.10%)	7.791	0.02
	46-100 scores	4 (4.20%)	25 (15.50%)	7 (14.90%)	36 (11.90%)		
PSQI	0-10 scores	85 (89.50%)	154 (95.70%)	45 (95.70%)	284 (93.70%)	3.826	0.156
	11-21 scores	10 (10.50%)	7 (4.30%)	2 (4.30%)	19 (6.30%)		
Cohesion	0-63 scores	42 (44.20%)	38 (23.60%)	9 (19.10%)	89 (29.40%)	15.034	0.001
	64-100 scores	53 (55.80%)	123 (76.40%)	38 (80.90%)	214 (70.60%)		
Adaptability	0-50 scores	77 (81.10%)	89 (55.30%)	19 (40.40%)	185 (61.10%)	26.648	0
	51-100 scores	18 (18.90%)	72 (44.70%)	28 (59.60%)	118 (38.90%)		
GAD-7	0-9 scores	76 (80.00%)	145 (90.10%)	44 (93.60%)	265 (87.50%)	7.438	0.024
	10-21 scores	19 (20.00%)	16 (9.90%)	3 (6.40%)	38 (12.50%)		
PHQ-9	0-9 scores	58 (61.10%)	133 (82.60%)	46 (97.90%)	237 (78.20%)	28.908	0
	10-27 scores	37 (38.90%)	28 (17.40%)	1 (2.10%)	66 (21.80%)		

0 was set as the reference category. The present study dichotomized all continuous scale scores according to validated clinical cut-offs of the instruments or widely accepted cut-offs for individuals with infertility, with consideration given to the score distribution in the current sample. This approach may help ensure both the clinical relevance and statistical appropriateness of the categorization.

### 3.2 Latent profile analysis and subgroup nomenclature of psychological resilience among individuals with recurrent implantation failure

**Figure 1:**
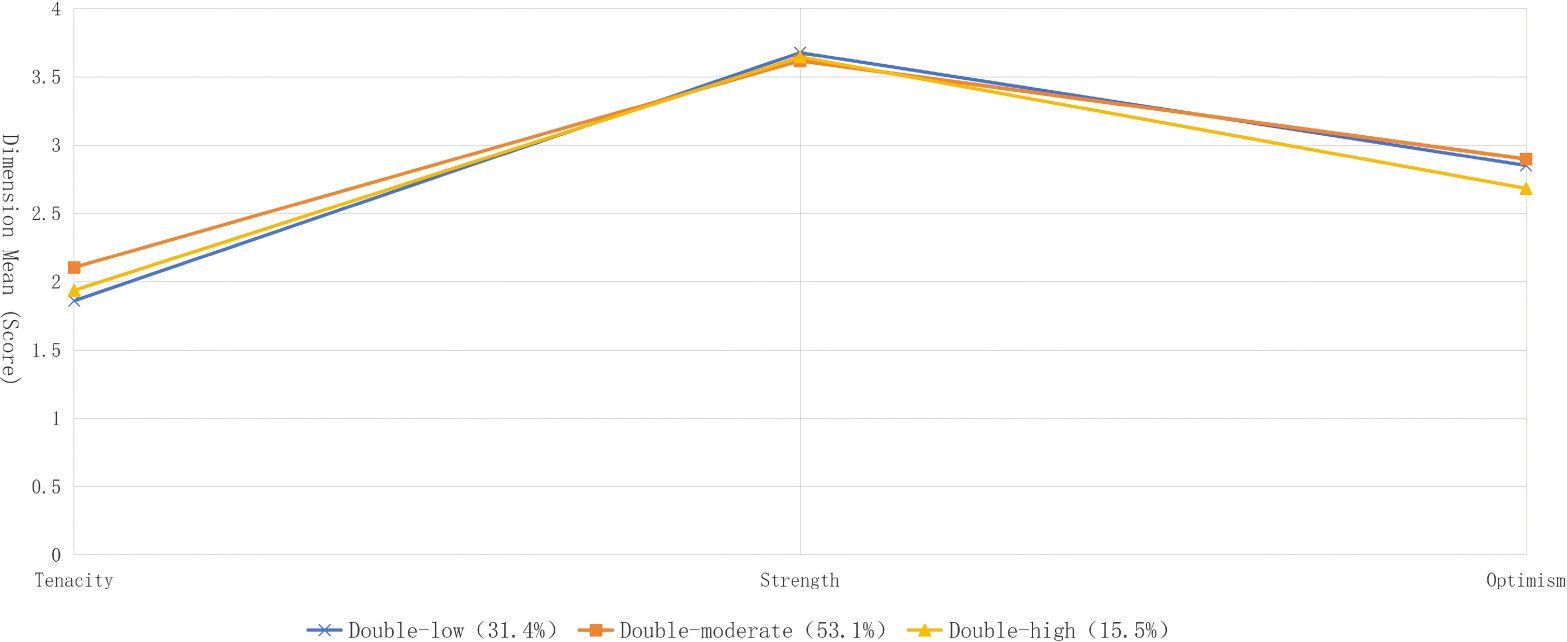
Characteristic distribution of the three psychological resilience latent profiles among individuals from couples with recurrent implantation failure.

Using the scores of the three dimensions of the Psychological Resilience Scale as manifest variables, latent profile models with 1 to 5 profiles were fitted successively for comparison. The findings indicated that as the number of latent profiles increased, the absolute value of Log (L) declined progressively, while AIC, BIC and aBIC also exhibited a decreasing trend, implying that models with more profiles yielded better fit. The 4-profile model achieved the highest entropy value; however, one of its subgroups accounted for merely 6.60% of the total sample, and the LMRT result (*P=0.0111*) failed to reach statistical significance. Based on a comprehensive evaluation of model fit, classification accuracy and subgroup rationality, the 3-profile model was determined to be the optimal latent profile model. The outcomes of model fitting are presented in **Table 2**. According to the results of the 3-profile classification, the latent profile characteristics of psychological resilience among individuals from couples with recurrent implantation failure were illustrated in this study, see **Figure 1**. The subgroups were named according to the scores of the two core dimensions (tenacity and strength) of the Psychological Resilience Scale: the low tenacity-low strength subgroup (double-low subgroup, n=95, 31.4%), the moderate tenacity-moderate strength subgroup (double-moderate subgroup, n=161, 53.1%), and the high tenacity-high strength subgroup (double-high subgroup, n=47, 15.5%).

**Table 2:** Latent profile model fit indices of psychological resilience among individuals from couples with recurrent implantation failure.

Model	Log(L)	AIC	BIC	ABIC	LMRT	BLRT	Entropy	Proportion
1	-2416.049	4844.098	4866.381	4847.352	/	/	/	/
2	-2341.782	4703.564	4740.701	4708.986	0.0001	0.0000	0.715	0.40594 0.59406
3	-2285.918	4599.836	4651.829	4607.428	0.0000	0.0000	0.841	0.31353 0.15512 0.53135
4	-2273.608	4583.216	4650.063	4592.977	0.0111	0.0000	0.852	0.06601 0.49175 0.29043 0.15182
5	-2267.135	4578.270	4659.972	4590.200	0.1955	0.1034	0.813	0.41584 0.06601 0.29703 0.12211 0.09901

### 3.3 Multivariate logistic regression analysis of latent profiles of psychological resilience among individuals with recurrent implantation failure

Independent variables that exhibited statistical significance in univariate analyses were stratified and appropriately consolidated to enhance the clarity and interpretability of regression outcomes. The coding scheme for independent variables is presented in **Table 3**. Subsequent multivariate Logistic regression analysis demonstrated that, when comparing the double-low group with the double-high group, male participants, those with high adaptability, and individuals with low depression severity were more prone to be categorized into the double-high group. When the double-low group was compared with the double-moderate group, participants with high adaptability and those with low depression severity were more likely to be assigned to the double-moderate group. In the comparison between the double-moderate group and the double-high group, male participants and individuals with low depression severity were more prone to be categorized into the double-high group. Multicollinearity testing was conducted for the multivariate logistic regression model in this study. Results demonstrated that the variance inflation factor (VIF) values of all independent variables were less than 3, with tolerance values greater than 0.3; these findings suggest an absence of multicollinearity within the model and support the stability and reliability of the regression results. The outcomes of multivariate Logistic regression analysis for psychological resilience latent profiles among individuals from couples with recurrent implantation failure (RIF) are presented in **Table 4**.

**Table 3:** Variable coding table.

Variables	Coding
Gender	0=Male;1=Female
SSRS	0 = 0-45 scores;1 = 46-100 scores
Family adaptability	0 = 0-50 scores;1 = 51-100 scores
Family cohesion	0 = 0-63 scores;1 = 64-100 scores
GAD-7	0 = 0-9 scores, 1 = 10-21 scores
PHQ-9	0 = 0-9 scores;1 = 10-27 scores

0 was set as the reference category.

**Table 4:** Multivariate logistic regression analysis of psychological resilience latent profiles among individuals from couples with recurrent implantation failure.

Variables		B	SE	Wald χ2	P	OR	95%CI
C1:C3^1^	Constant	-1.830	1.317	1.931	0.165		
	Gender	1.145	0.406	7.969	0.005	3.143	1.419-6.959
	SSRS	-0.740	0.698	1.124	0.289	0.477	0.121-1.874
	Cohesion	-0.342	0.515	0.442	0.506	0.710	0.259-1.948
	Adaptability	-1.338	0.465	8.290	0.004	0.262	0.105-0.652
	Anxiety	-0.615	0.798	0.594	0.441	0.541	0.113-2.583
	Depressive	3.272	1.109	8.711	0.003	26.373	3.002-231.702
C1:C2^1^	Constant	1.452	0.708	4.202	0.040		
	Gender	0.360	0.304	1.404	0.236	1.433	0.79-2.6
	SSRS	-1.014	0.576	3.097	0.078	0.363	0.117-1.122
	Cohesion	-0.414	0.321	1.667	0.197	0.661	0.353-1.239
	Adaptability	-0.841	0.344	5.968	0.015	0.431	0.22-0.847
	Anxiety	0.017	0.449	0.001	0.970	1.017	0.422-2.451
	Depressive	0.821	0.348	5.552	0.018	2.272	1.148-4.495
C2:C3^2^	Constant	-3.281	1.204	7.431	0.006		
	Gender	0.785	0.345	5.171	0.023	2.193	1.115-4.313
	SSRS	0.274	0.486	0.317	0.573	1.315	0.507-3.411
	Cohesion	0.072	0.490	0.021	0.883	1.075	0.411-2.809
	Adaptability	-0.497	0.397	1.570	0.210	0.608	0.28-1.324
	Anxiety	-0.632	0.764	0.684	0.408	0.531	0.119-2.377
	Depressive	2.452	1.101	4.963	0.026	11.609	1.343-100.36

C1, Double-low subgroup; C2, Double-moderate subgroup; C3, Double-high subgroup; 1, The double-low group was used as the reference group; 2, The double-moderate group was used as the reference group; 3, All regression models were tested for multicollinearity, with the variance inflation factor (VIF) of all independent variables < 3 and Tolerance > 0.3, indicating no multicollinearity.

## 4 Discussion

### 4.1 Psychological resilience among individuals from couples with recurrent implantation failure

The total psychological resilience score of 303 individuals from couples with recurrent implantation failure (RIF) undergoing assisted reproductive technology (ART) was (26.66 ± 6.319). Based on the grading criteria of the CD-RISC-10 scale (with a total score range of 0–40, where higher scores indicate stronger psychological resilience), the overall psychological resilience of RIF individuals was at a moderate level. In contrast, Wei et al. (22)reported that the total psychological resilience score of individuals with first implantation failure was (27.58 ± 6.34), which was higher than that of RIF individuals. This finding suggests that the experience of recurrent implantation failure may exert a measurable impact on psychological resilience among individuals from affected couples, which underscores the need for targeted clinical interventions and attention. From the individual level, the present study provides a foundation for subsequent dyadic resilience-matching research among couples with recurrent implantation failure and sheds light on the potential interaction mechanism of resilience between partners.

### 4.2 Group heterogeneity in psychological resilience among individuals from couples with recurrent implantation failure

The present study detected group heterogeneity in psychological resilience levels among individuals with recurrent implantation failure via latent profile analysis, classifying the participants into three distinct subgroups. The double-low subgroup constituted 31.4% of the total sample. Individuals in this subgroup tended to discontinue treatment following implantation failure, demonstrated poor frustration tolerance, lacked the capacity for autonomous emotional regulation after experiencing emotional distress, and possessed inadequate internal coping resources. The double-moderate subgroup made up 53.1% of the cohort. These individuals would experience temporary hesitation after implantation failure yet refrain from abandoning treatment, and they relied on encouragement from healthcare providers and family members to regain confidence and proceed with therapy. The double-high subgroup represented 15.5% of the participants. Despite encountering multiple episodes of recurrent implantation failure, these individuals actively communicated with healthcare professionals to modify treatment strategies and persisted in completing the entire treatment course. This classification outcome first indicates that clinical interventions should be stratified and tailored precisely. For the double-low subgroup, these individuals should be prioritized as key intervention targets, with urgent psychological assessment and crisis intervention implemented immediately after implantation failure. Clarification of factors linked to implantation failure, assignment of dedicated healthcare providers, and provision of family support training may be associated with the mitigation of deficits in internal coping resources, as well as a potential reduction in the likelihood of treatment discontinuation among individuals from couples with RIF. For the double-moderate subgroup, intervention should be centered on guidance. Personalized feedback should be delivered at critical treatment junctures to enhance confidence. In the meantime, a peer support platform should be constructed to facilitate the acquisition of coping skills from the high-resilience subgroup, thus preventing the regression to a low-resilience state. The double-high subgroup can serve as a valuable resource for psychological intervention. Through experience sharing and peer exchanges, they can exert a positive psychological influence on individuals with low psychological resilience and fulfill a demonstration and leading role.

### 4.3 Analysis of influencing factors for latent subgroups of psychological resilience among couples with recurrent implantation failure

#### 4.3.1 Gender

The regression results indicated that male individuals were more likely to be classified into the high-resilience group. It is speculated that the underlying reason for this difference may lie in the fact that women, as the direct recipients of assisted reproductive treatment, have to undergo a series of physical procedures including ovulation induction, oocyte retrieval, and embryo transfer. Physical discomfort and the uncertainty of the treatment process may exacerbate psychological stress; On the other hand, the implicit social and cultural perception that “reproductive responsibility falls more on women” may cause women to experience more self-denial and external criticism after implantation failure, thereby reducing their psychological resilience. In contrast, men tend to adopt behavioral coping strategies such as actively communicating treatment plans to deal with failure, rather than expressing vulnerability through emotional catharsis. This finding is consistent with the research conclusion of Kong (23) that infertile women are more prone to emotional distress during assisted reproductive treatment. The fact that male individuals are more likely to be classified into the high-resilience group does not mean that they are free from psychological stress. Traditional perceptions often overlook the psychological needs of men in reproductive treatment. The results of this study suggest that clinical practice should pay attention to the hidden psychological risks of male individuals. Meanwhile, high-resilience male individuals can be encouraged to participate in peer support programs, using their behavioral coping experiences to set an example for other individuals.

#### 4.3.2 Family adaptability

The regression results indicated that higher family adaptability was significantly associated with the transition from the double-low class to the double-moderate and double-high classes, and may serve as a key protective correlate of psychological resilience. This suggests that the flexible adjustment capacity of the family system, such as effective family communication following implantation failure and collaborative engagement with treatment regimens, may effectively buffer the impact of reproductive setbacks, provide individuals with stable emotional support and practical resources, and thus be related to higher levels of psychological resilience. Clinical interventions may need to move beyond a sole focus on the individual patient (24). It may be beneficial to guide family members in developing effective communication strategies after implantation failure, establish flexible coping plans for addressing treatment-related challenges, and support the development of family functioning as a protective mechanism for psychological resilience. In particular, targeted interventions for families with low adaptability may help promote a shift from the double-low class toward moderate or high resilience profiles.

#### 4.3.3 Depression

The regression results indicated that the association of depression severity was the most prominent. A low-depression state was significantly correlated with the transition from the double-low group to the double-moderate group and from the double-moderate group to the double-high group, which is highly consistent with the conclusions of previous studies suggesting that “depressive emotions are associated with reduced psychological resilience, potentially through depleting psychological resources and weakening the ability to cope with adversity” (23, 25, 26). Recurrent implantation failure may keep individuals from affected couples in a prolonged “expectation-disappointment” negative cycle, which may increase vulnerability to depressive symptoms such as self-doubt; meanwhile, concerns about the uncertainty of treatment outcomes may be associated with anxiety symptoms. These negative emotions are likely to form a vicious circle of “emotional problems → reduced psychological resilience→higher susceptibility→emotional problems”.Depressive emotions may directly negate an individual’s coping resources, weaken their courage to face failure, and ultimately be related to a lower level of psychological resilience. This result is mutually corroborated with the conclusions of Gao et al. and Zhang et al. (26, 27). Addressing emotional problems may be crucial for individuals with recurrent implantation failure. Clinical interventions could involve full-course dynamic monitoring of psychological depression during treatment, promptly initiate professional interventions such as cognitive behavioral therapy for individuals with positive depression, conduct psychiatric consultation when necessary, and lay a potential foundation for the improvement of psychological resilience by alleviating depressive symptoms.

### 4.4 Sleep quality and social support

This study found that social support (SSRS) was not identified as an independent factor associated with psychological resilience among individuals with RIF. This observation may be related to the specific characteristics of support needs in the RIF population. Compared with external social support from friends, colleagues, and other non-family members, emotional support between spouses and within the family may exert a more direct regulatory association with their psychological resilience. This suggests that subsequent studies can further distinguish the effects of different types of support. However, no statistically significant difference was observed in sleep quality (PSQI) across different psychological resilience latent profiles among individuals with RIF. This finding may be related to the relatively good overall sleep quality of the study sample. Potential skewness in the sample distribution may have contributed to the non-significant statistical results. Future studies may need to expand the sample size, incorporate individuals with RIF who have poor sleep quality, and conduct further verification to clarify this association.

## 5 Conclusion

This study identified the characteristics and core factors associated with psychological resilience among individuals from couples with recurrent implantation failure. Their overall psychological resilience was at a moderate level, with significant population heterogeneity, which could be categorized into three latent profiles: low tenacity-low strength group, moderate tenacity-moderate strength group, and high tenacity-high strength group. Multivariate Logistic regression confirmed that gender, family adaptability, and depression severity were independent core factors associated with the affiliation of psychological resilience profiles. Males were more likely to be classified into the high-resilience group, while high family adaptability was associated with a reduced likelihood of being in a low-resilience state, and greater depression severity was significantly correlated with an increased probability of falling into a low-resilience state. Clinically, stratified interventions may be implemented for different psychological resilience profiles: the low-resilience group should focus on depression intervention and family support training, the moderate-resilience group may benefit from strengthened empowerment guidance, and the high-resilience group could play an exemplary role. Meanwhile, psychological resilience assessment and depression screening may be incorporated into routine diagnosis and treatment. By enhancing family functions, alleviating negative emotions, and constructing support networks, it may be possible to improve individuals’ psychological resilience and treatment adherence, thereby potentially promoting their mental health and treatment prognosis.

However, this study still has certain limitations. For example, the sample was derived from a single center, which may introduce selection bias; the cross-sectional survey design was adopted, which cannot establish the causal relationship between psychological resilience and various factors; the sample size was relatively limited, and the potential associations of some actors may not have been fully explored. Future studies may expand the sample size, conduct multi-center studies, and adopt longitudinal designs to explore the trajectory changes and influencing factors of psychological resilience, so as to provide more sufficient evidence support for the formulation of personalized psychological intervention programs.

## Data Availability

The original contributions presented in the study are included in the article/supplementary material. Further inquiries can be directed to the corresponding author.
