# Correction: Heterogeneity of psychological resilience among individuals with recurrent implantation failure: a latent profile analysis

**DOI:** 10.3389/fpsyt.2026.1850097

**Published:** 2026-04-21

**Authors:** Yue Peng, Yan Pu, Yuyang Wang, Shiya Li, Yan Wang, Jinbo Fang

**Affiliations:** 1 Department of Reproductive Medicine Nursing, West China Second University Hospital, Sichuan University/West China School of Nursing, Sichuan University, Chengdu, China; 2 Key Laboratory of Birth Defects and Related Diseases of Women and Children (Sichuan University), Ministry of Education, Chengdu, China; 3 West China School of Nursing, Sichuan University, Chengdu, China

**Keywords:** assisted reproductive technology, clinical decision, latent profile analysis, mental health, psychological resilience, recurrent implantation failure

Author Yue Peng was erroneously assigned to affiliation 3 “West China School of Nursing, Sichuan University, Chengdu, China”. This affiliation has now been removed for author Yue Peng.

Author Shiya Li was erroneously assigned to affiliation 1 “Department of Reproductive Medicine Nursing, West China Second University Hospital, Sichuan University/West China School of Nursing, Sichuan University, Chengdu, China”. This affiliation has now been removed for author Shiya Li.

Affiliation 1. was erroneously given as “Department of Reproductive Medicine Nursing, West China Second University Hospital, Sichuan University, Chengdu, China”. This should read:

“Department of Reproductive Medicine Nursing, West China Second University Hospital, Sichuan University/West China School of Nursing, Sichuan University, Chengdu, China”.

The original version of this article has been updated.

